# Cluster analysis of flowcytometric immunophenotyping with extended T cell subsets in suspected immunodeficiency

**DOI:** 10.1002/iid3.1106

**Published:** 2023-12-06

**Authors:** Luca Seitz, Daniel Gaitan, Caroline M. Berkemeier, Christoph T. Berger, Mike Recher

**Affiliations:** ^1^ Immunodeficiency Laboratory, Department of Biomedicine University Hospital Basel and University of Basel Basel Switzerland; ^2^ Department of Rheumatology and Immunology, Inselspital, University Hospital Bern University of Bern Bern Switzerland; ^3^ Division of Medical Immunology, Laboratory Medicine University Hospital Basel Basel Switzerland; ^4^ University Center for Immunology University Hospital Basel Basel Switzerland; ^5^ Translational Immunology, Department of Biomedicine University of Basel Basel Switzerland

**Keywords:** cluster analysis, flowcytometry, inborn errors of immunity, primary immunodeficiency, T cell subsets

## Abstract

**Background:**

Patients with immunodeficiencies commonly experience diagnostic delays resulting in morbidity. There is an unmet need to identify patients earlier, especially those with high risk for complications. Compared to immunoglobulin quantification and flowcytometric B cell subset analysis, expanded T cell subset analysis is rarely performed in the initial evaluation of patients with suspected immunodeficiency. The simultaneous interpretation of multiple immune variables, including lymphocyte subsets, is challenging.

**Objective:**

To evaluate the diagnostic value of cluster analyses of immune variables in patients with suspected immunodeficiency.

**Methods:**

Retrospective analysis of 38 immune system variables, including seven B cell and sixteen T cell subpopulations, in 107 adult patients (73 with immunodeficiency, 34 without) evaluated at a tertiary outpatient immunology clinic. Correlation analyses of individual variables, k‐means cluster analysis with evaluation of the classification into “no immunodeficiency” versus “immunodeficiency” and visual analyses of hierarchical heatmaps were performed.

**Results:**

Binary classification of patients into groups with and without immunodeficiency was correct in 54% of cases with the full data set and increased to 69% and 75% of cases, respectively, when only 16 variables with moderate (*p* < .05) or 7 variables with strong evidence (*p* < .01) for a difference between groups were included. In a cluster heatmap with all patients but only moderately differing variables and a heatmap with only immunodeficient patients restricted to T cell variables alone, segregation of most patients with common variable immunodeficiency and combined immunodeficiency was observed.

**Conclusion:**

Cluster analyses of immune variables, including detailed lymphocyte flowcytometry with T cell subpopulations, may support clinical decision making for suspected immunodeficiency in daily practice.

## Graphical abstract

Patients with immunodeficiencies, including primary immunodeficiencies, commonly experience diagnostic delays resulting in morbidity. There is an unmet need to identify such patients earlier and assign a correct diagnosis and personalized prognosis. Our results support the use of detailed lymphocyte flowcytometry, including extended T cell subpopulations, in the routine work‐up for suspected immunodeficiency to achieve these goals.

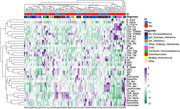

## 1 INTRODUCTION

The knowledge of genetically determined diseases of the immune system has increased tremendously in the last two decades, with almost 500 entities being recognized today.^1^ Immunodeficiencies (IDs) are heterogeneous diseases characterized by reduced immunocompetence. They can be classified into primary ID (PID), which is genetically determined, and secondary ID, which is driven by exogenous factors such as immunosuppressants or malignancy.^2^ Also, secondary ID has been demonstrated in patients with yet undiagnosed PID.^3^ In addition to increased susceptibility to infections, PID patients often have associated autoimmunity, autoinflammation and/or polyclonal lymphoproliferation (enlarged spleen and lymph nodes). Therefore, the term “inborn errors of immunity” (IEI) is now often preferred over PID.^1^, ^4^ The reduced costs of next‐generation sequencing has paved the way for the rapid increase in known entities of IEI, with the bottleneck now being the thorough validation of the many variants of unknown significance identified.^5^ Secondary IDs are also becoming more prevalent, mainly due to increases in targeted immune‐modulating treatments and clinical prescriptions. Timely diagnosis of ID and targeted immune‐modulation by an increasing array of available pharmacotherapeutics, such as Janus‐kinase‐inhibitors, can prevent complications and improve quality of life.^6^, ^7^ ID and IEIs remain underdiagnosed, especially in adults.^8^, ^9^ While genetic diseases are considered promptly in children, this is often not the case in adults. This could be due to a lack of awareness of late presentations of IEI, multisystemic manifestations of IEIs or the growing number of adult‐onset phenocopies of IEI due to cytokine‐neutralizing autoantibodies or somatic mutations.^10^, ^11^ Clinical scoring systems as diagnostic aids have been developed and diagnostic algorithms proposed, but diagnostic accuracy remains unsatisfactory.^12^, ^13^ While for the most frequent IEI category in adults, IEI with predominantly antibody deficiencies, the screening tests seem relatively straightforward (immunoglobulin levels, response to vaccinations), the diagnostic procedures for other categories of IEI are more complex.^12^ Also, most genetic studies in PID have a limited diagnostic yield, varying between 15% and 30%, with lower yields in adults.^14^, ^15^, ^16^ Lymphocyte flowcytometry (LFC) of peripheral blood is a readily available diagnostic tool and is becoming increasingly important in diagnosing IEI.^17^, ^18^, ^19^, ^20^ The results of a recent Egyptian study of more than a thousand pediatric patients support the value of LFC for IEI diagnosis: with a very detailed LFC protocol, including protein expression assays, 73% of patients with an IEI could be diagnosed.^17^ Reference values for lymphocyte subsets for different age groups have been published.^20^, ^21^, ^22^, ^23^, ^24^, ^25^ Elevation or specific absence of lymphoid cells or proteins within cells detected by flowcytometry of peripheral blood can sometimes suggest a specific IEI entity. Examples include autoimmune lymphoproliferative syndrome (ALPS) or specific forms of hereditary hemophagocytic lymphohistiocytosis such as perforin deficiency.^19^, ^26^ As LFC is expensive, an initial typical screening panel in most clinics is limited to an enumeration of natural killer (NK) cells, B cells (often including memory B cells) and T cells with limited subsets (i.e., total CD4+ and CD8+ T cells). Further differentiation of T cells into functional subpopulations is only performed routinely in a minority of PID centers. International efforts have been made to standardize extended LFC investigations in suspected lymphoid PID (EuroFlow Consortium, PID Orientation Tube [PIDOT]), allowing comparison of results between centers.^20^, ^27^ While PIDOT (identifying >20 blood leukocyte (sub)populations with 12 markers), is undoubtedly a significant step forward in the diagnostics of PID and IEI, it is limited to laboratories that have implemented its use. Also, its algorithm does not include other non‐flowcytometric immune variables typically measured during work‐up of ID, such as immunoglobulins.^27^ Measurement and correlation of T cell subpopulations with other immune system markers in routine daily practice remains scarce.^27^, ^28^ To what extent T cell subpopulations correlate with other laboratory variables, such as serum immunoglobulins, myeloid cells, and B cell subpopulations and whether these variables are of redundant or additive value for disease classification (presence or absence of ID), remains ill defined. To further investigate these aspects, we evaluated T cell subpopulations in the peripheral blood of 107 individuals and correlated them with each other, with age, and with a complete set of routine immune‐laboratory variables. Unsupervised clustering was used to evaluate multiple variables, clinical ID sub‐entities and the classification into immunodeficient versus immunocompetent individuals.

## 2 METHODS

The study was approved by the local institutional review board (ethics committee Nordwest‐ und Zentralschweiz, Switzerland, approval number 2017‐02029).

### 2.1 Study population

The following individuals were consecutively included in this retrospective analysis: adult individuals assessed at the Immunodeficiency Clinic of the University Hospital Basel, Switzerland, between November 2015 and November 2017, in whom peripheral blood T cell subsets had been analyzed and who gave written informed consent. The following data were collected retrospectively by individual chart review: clinical diagnosis, age, sex and immunologic laboratory variables. In patients receiving immunoglobulin replacement therapy, IgG and IgG subclass values before its start were used for analysis. The clinical diagnosis was determined according to the ESID definitions, where available, by the last author (MR), who has extensive experience with IEI and ID. A total of 107 individuals were identified: 34 (32%) were considered immunocompetent (No‐ID), and 73 (68%) were given a diagnosis of ID. The ID group consisted of four subgroups containing ≥ 5 individuals: selective IgG subclass deficiency (*n* = 29); common variable immunodeficiency (*n* = 10); combined immunodeficiency (*n* = 8) and autoinflammation (*n* = 8). The remaining diagnostic entities were multiple autoimmunity without antibody deficiency (*n* = 4); selective IgA deficiency (*n* = 3); secondary antibody deficiency (*n* = 2); T‐cell large granular lymphocytic leukemia, stiff person syndrome, persistent polyclonal B cell lymphocytosis, monoclonal B cell lymphocytosis, isolated CD4+ lymphopenia, monoclonal gammopathy, autoimmune lymphoproliferative syndrome, antibody deficiency (not further specified), and immunodeficiency (not further specified) (each *n* = 1 [9 in total]). More than 90% (66/73) of patients in the ID group were classified as PID/IEI. Age was equally distributed between the groups (mean age 43.5 (No‐ID) and 45.6 (ID)) and there were 59% versus 71% female individuals in the No‐ID versus ID groups, respectively.

### 2.2 Flowcytometry and gating strategy

LFC was performed as part of the initial diagnostic workup. T cell subpopulations were characterized using a sequential gating strategy (Figure E1). In a first step, single events were identified using FSC‐H/FSC‐A profile. Further analysis of lymphocytes was done using a combination of CD45 expression and SSC/FSC properties. Within the CD45+ cells, T cells were defined as CD3+ cells and subdivided into CD4+ and CD8+ T cells based on the expression of the respective marker. Presence of CCR7, CD45RA or CD45RO was used to further discriminate CD8+ and CD4+ T cells into the following T cells subpopulations; CD8_Naïve (CD45RA+, CD45RO−, CCR7+), CD8_CM (CD45RA−, CD45RO+, CCR7+), CD8_EM (CD45RA−, CD45RO+, CCR7−), CD8_TEMRA (CD45RA+, CD45RO−, CCR7−) and CD4_Naïve (CD45RA+, CD45RO−, CCR7+), CD4_CM (CD45RA−, CD45RO+, CCR7+), CD4_EM (CD45RA−, CD45RO+, CCR7−), CD4_TEMRA (CD45RA+, CD45RO−, CCR7−). Within the CD4+ T cells, CD25 and CD127 profiles were used for identification of CD4_Treg (CD25+ CD127 low). Within the CD4+ CD45RA+ CD45RO− T cells, CD31 and CXCR5 surface expression were used to characterize CD4_RTE (CD45RA+, CD45RO−, CD31+) or CD4_FH (CD45RA+, CD45RO−, CCR7+, CXCR5+), respectively. Activated CD4+ and CD8+ T cells were identified based on the expression of HLA‐DR (CD4_act [CD4+ HLA‐DR+] and CD8_act [CD4− HLA‐DR+]). B cells were defined as CD19+ cells within the CD45+ cells. Using surface expression profiles of IgM, IgD, CD27 and CD38, B cell subpopulations were further discriminated into the following entities: B_Naïve (IgD+, CD27−), B_MZ (IgD+ CD27+), B_Mem (IgD−, IgM−, CD27+), B_Trans (IgM+++, CD38+), B_Plasm (IgM−, CD38 + ), CD21_low (CD21low, CD38−). (Table 1).

**Table 1:** Subsets of T and B cells.

T cells			B cells		
Subset CD4+	Abbreviation*	Cell surface marker	Subset	Abbreviation*	Cell surface marker
Naïve	CD4_Naïve	CD4+, CD45RA+, CD45RO−, CCR7+	Naïve	B_Naïve	IgD+, CD27−
Central memory	CD4_CM	CD4+, CD45RA−, CD45RO+, CCR7+	Transitional	B_Trans	IgM+++, CD38+
Effector memory	CD4_EM	CD4+, CD45RA−, CD45RO+, CCR7−	Plasmablast	B_Plasm	IgM−, CD38+
Terminally differentiated	CD4_TEMRA^1^	CD4+, CD45RA+, CD45RO−, CCR7−	CD21_low	B_CD21lo	CD21 low, CD38−
Recent thymic emigrants	CD4_RTE	CD4+, CD45RA+, CD45RO−, CCR7+, CD31+	Marginal zone	B_MZ	IgD+, CD27+
Follicular helper	CD4_FH	CD4+, CD45RA−, CD45RO+, CXCR5+	Memory	B_Mem	IgD−, IgM−, CD27+
Activated	CD4_Act	CD4+, HLA‐DR+			
Regulatory	CD4_Treg	CD4+, CD25+, CD127 low			
**Subset CD8**+					
Naïve	CD8_Naïve	CD8+, CD45RA+, CD45RO−, CCR7+			
Central memory	CD8_CM	CD8+, CD45RA−, CD45RO+, CCR7+			
Effector memory	CD8_EM	CD8+, CD45RA−, CD45RO+, CCR7−			
Terminally differentiated	CD8_TEMRA	CD8+, CD45RA+, CD45RO−, CCR7−			
Activated	CD8_Act	CD8+, HLA‐DR+			

*Note*: *This is the abbreviation used in the figures and the text.

Abbreviation: TEMRA, T cell effector memory re‐expressing CD45RA.

### 2.3 Statistics

R version 4.2.0 (R Core Team, 2022) was used for all analyses and plots. Laboratory values below the lower limit of detection (LOD) for a given marker were set to LOD/2 for the analysis. For each laboratory immune variable and age, the Wilcoxon rank‐sum test was used to compare the No‐ID and the ID groups; *p* values of <.1, <.05, and <.01 were interpretated as evidence for weak, moderate, and strong difference between groups. Age and a total of 38 different laboratory variables were analyzed. In addition to the 19 T and B cell subclasses described in Table 1, the following 19 variables were analyzed: absolute cell counts of total T cells, CD4+ and CD8+ T cells, total B cells, NK cells, leukocytes, neutrophils, lymphocytes, monocytes, eosinophils, and basophils; IgG, IgA, IgM, IgE and IgG subclasses (IgG1, IgG2, IgG3, IgG4). We included relative (percentage) and absolute values for five variables (total T‐cells, CD4+ T cells, CD8+ T cells, total B cells, and NK cells) only for the correlation maps and boxplots. For cluster analyses, only the absolute values of these five variables were considered to include each cell type/subclass only once. Spearman's r was used for the correlation of variables. The strength of correlation was interpreted as follows (correlation coefficient r): +/− 0 – 0.19 = very weak, +/− 0.2 – 0.39 = weak, +/− 0.4 – 0.59 = moderate, +/− 0.6 – 0.79 = strong, +/− 0.8 – 1 = very strong.^29^

For cluster analysis of 37 laboratory variables, missing values (176/3959 = 4.4%) were imputed by nonparametric imputation using random forests. Because IgE values were missing in 37% of patients, IgE was excluded from the cluster analysis. Clustering tendency was assessed with the Hopkins statistics (values close to 1 demonstrate nonuniform distribution) for each data set separately.^30^ The average Silhouette width method was used to estimate the optimal number of clusters for each data set separately.^30^ Different clustering methods were used for two main different objectives.

K‐means clustering was performed to assess whether grouping patients into clusters of ID versus No‐ID is possible based on different sets of immune variables. The corrected (adjusted) Rand index was calculated to quantify the agreement of the k‐means clustering with the clinical classification (ID vs. No‐ID) (range: −1 (no agreement) to 1 [perfect agreement]).^30^ The McNemar's test was used to compare the correct allocation into two clusters (ID vs. No‐ID) between different sets of variables (*p* < .05 regarded as significant). K‐medoids clustering with the “partitioning around medoids” algorithm was used in a sensitivity analysis because it is robust to outliers.^30^

Hierarchical clustering was performed to allow for visual analysis of the data. For cluster heatmaps, scaling was applied to rows, rows were clustered using correlation distance and complete linkage, and columns were clustered using Euclidean distance and complete linkage.^30^ The cluster heatmaps were visually assessed and the height for cutting the dendrograms chosen accordingly. The distribution of No‐ID versus ID cases and clustering of the ID subentities e.g., IgA deficiency, was also assessed visually. Principal components analysis (PCA) was used for an additional descriptive visualization of clusters from the hierarchical clustering. For hierarchical clustering, the agreement with the clinical classification (No‐ID vs. ID) was not assessed statistically.

## 3 RESULTS

For 7/43 (16%) laboratory immune variables there was strong evidence (*p* < .01) for a difference between the No‐ID versus the ID group: total T cells, total CD4+ T cells (per µl); B_Mem (percentage of total B cells); IgG, IgG2, IgG4, IgA (g/L). For additional 11 variables, there was at least moderate evidence for a difference (*p* < .05) (total of 18/43 [42%] variables): total T cells (percentage of lymphocytes); CD4_Naïve, CD4_EM, CD4_RTE, CD4_Act (all percentage of total CD4+ T cells); total CD8 + T cells (per µL); total lymphocytes, basophils (per L); IgG1, IgG3 (g/L); IgE (IU/ml). For additional five variables, there was at least weak evidence for a difference (*p* < .1) (total of 23/43 (53%) variables): total CD4+ T cells (percentage of lymphocytes); CD4_FH, CD8_Naïve (percentage of total CD4+ or CD8+ T cells); B_Naïve, B_Plasm (percentage of B cells). Although 7 of the 18 moderately differing variables (39%) are immunoglobulins, only for one B cell variable moderate evidence for a difference between groups was found. This is contrasted by eight T cell variables, including 6 T cell subsets (5 × CD4+, 1 × CD8+), demonstrating moderate evidence (*p* < .05) for a different distribution. Descriptive statistical values for each of the 43 laboratory variables and age are listed in Table E1 and Table E2, depicted as boxplots for each variable separately for both diagnostic groups.

**Figure 1:**
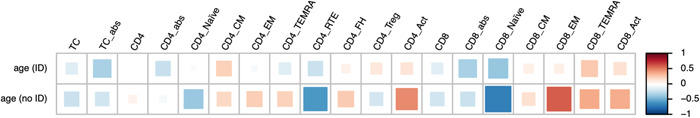
Correlation heatmap of T cell subsets and age. abs, absolute; ID, immunodeficiency; TC, T cells. Abbreviations for T cell subsets see Table 1. Color scale: Spearman's correlation coefficient r (−1 to 1).

For the No‐ID group, the following correlations of age versus T cell subsets and total T cells were notable (Figure 1): for CD4_RTE and CD8_Naïve, a moderate to strong negative correlation (r = − 0.57 and − 0.69), for CD4_Naïve a weak negative correlation (r = − 0.37) and a moderate and strong positive correlation for CD4_Act and CD8_EM (r = .48 and 0.6). Further correlations of T cell subsets and other immune system variables are shown in Figure E2–E7 and summarized in Text E1.

Cluster analyses were used to examine multivariate relationships. The dichotomous classification of No‐ID and ID patients into clusters was examined for multiple sets of variables (Table 2). Hopkins H showed clusterable data for each set using a threshold of 0.5. The optimal number of clusters was two for all datasets except for a set containing only B cell variables. Compared with 58/107 (54.2%) for the full data set, datasets limited to moderately and strongly differing variables correctly classified 74/107 (69.1%) and 80/107 (74.8%) of patients, respectively, which was significantly better for the latter. One cluster contained 91% and 96% ID patients for the datasets including moderately or strongly differing variables, respectively. This indicates that at least for a subset of ID patients, a very good separation was achievable. For the two best‐performing data sets, exclusion of T cell variables was assessed and compared (Table 2). The proportions were numerically slightly lower, but not reaching statistical significance compared to the data set including T cell subpopulations. Exploratively, data sets without the seven patients with likely secondary ID were also assessed; there was no relevant difference to the full data set (Table 2, last to rows). Again, a significantly better classification was found when only strongly differing variables were included. The results from the sensitivity analysis of k‐means versus k‐medoids are shown in Table E3. As the proportions for correct binary classifications were very similar, k‐medoids was not pursued further.

**Table 2:** Overview of k‐means cluster analysis of different data sets.

Patients included	Variables included	Hopkins H	Optimal number of clusters^a^	No‐ID/ID cases per cluster	Correctly classified	Corrected Rand index	McNemar's test (*p* value)
All 107	All 37 variables	0.827	2	C1 33/57 C2 1/16	54.2%	−0.06	n.a.
All 107	20 (with weak evidence for difference between groups [*p* < .1])	0.733	2	C1 3/30 C2 31/43	57.0%	−0.01	.834^b^
All 107	16 (with moderate evidence for difference between groups [*p* < .05])	0.827	2	C1 4/44 C2 30/29	69.1%	0.14	.085^b^
All 107	10 (with moderate evidence for difference between groups [*p* < .05], but no T cell subsets)	0.701	2	C1 2/42 C2 32/32	68.2%	0.12	.106^b^ 1.0^c^
All 107	7 (with strong evidence for difference between groups [*p* < .01])	0.703	2	C1 2/48 C2 32/25	74.8%	0.24	.014^b^
All 107	5 (with strong evidence for difference between groups [*p* < .01], but no T cell subsets)	0.758	2	C1 3/48 C2 31/25	73.8%	0.22	.015^b^ 1.0^c^
All 107	7 (only B cell variables)	0.908	4	C1 12/19; C2 17/2; C3 5/32; C4 0/1	n.a.	n.a.	n.a.
All 107	16 (only T cell variables)	0.723	2	C1 2/25 C2 32/48	53.3%	−0.03	1.0^b^
100 (no secondary)	All 37 variables	0.714	2	C1 33/52 C2 1/14	53.0%	−0.05	n.a
100 (no secondary)	7 (with strong evidence for difference between groups [*p* < .01])	0.726	2	C1 2/44 C2 32/22	76.0%	0.26	.006^d^

Abbreviations: C, cluster; ID, immunodeficiency; n.a., not applicable.

^a^ As defined by average Silhouette method.

^b^ Compared to data set with all 107 patients and all 37 variables.

^c^ Compared to data set including the T cell subsets.

^d^ Compared to data set with 100 patients and all 37 variables.

**Figure 2:**
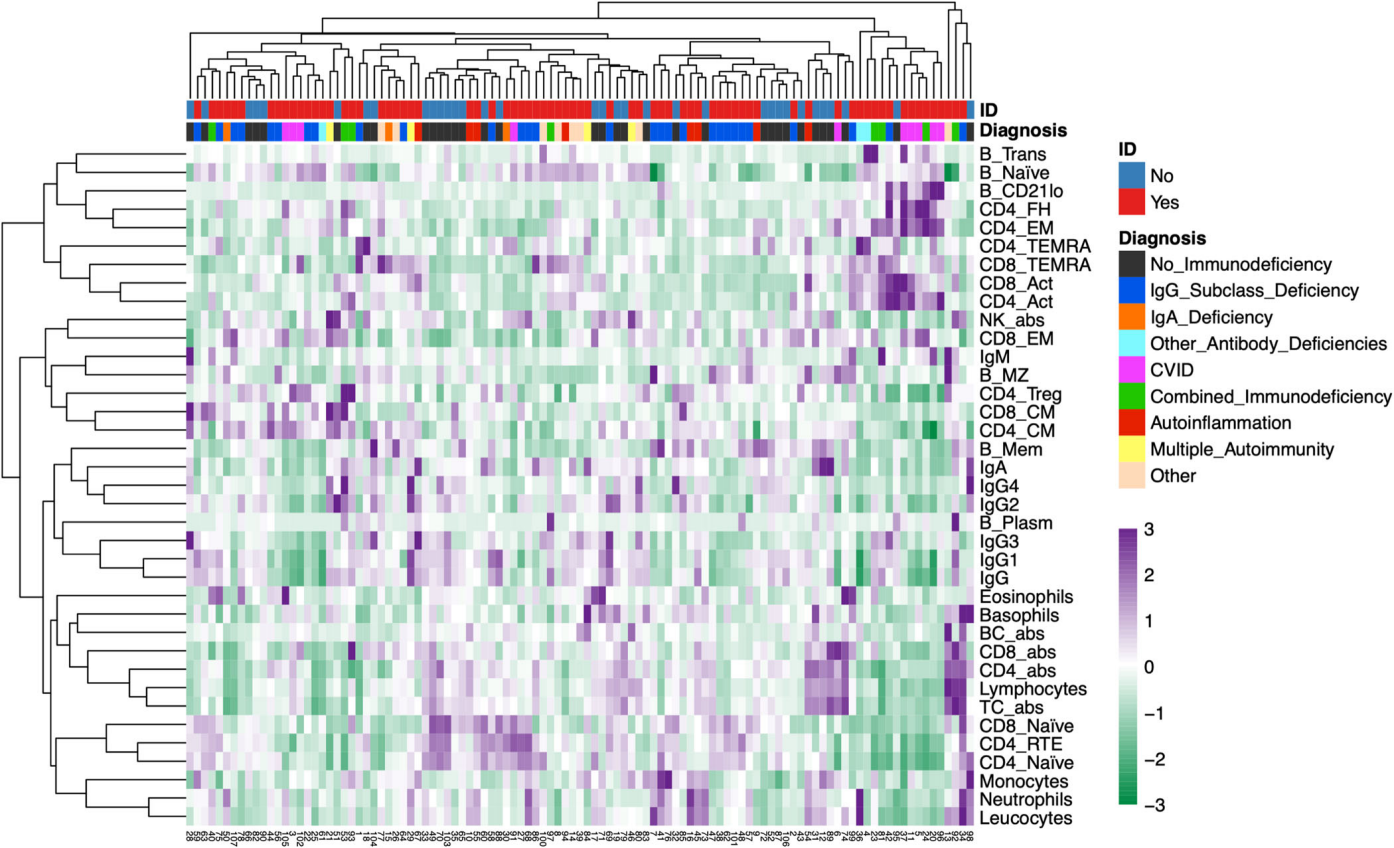
Cluster analysis of 37 laboratory variables from 107 patients with and without immunodeficiency: abs, absolute; CVID, common variable immunodeficiency; ID, immunodeficiency; NK, natural killer; TC, T cells. Abbreviations for B and T cell subsets see Table 1. Color scale: Z‐scores.

Hierarchical cluster heatmaps were visually analyzed to simulate a possible scenario for clinicians in terms of time and effort in daily practice. The full data set showed limited clustering of No‐ID versus ID cases and of ID subentities. (Figure 2) The red and blue categories appear to be distributed randomly, and apart from CVID and CID patients with high Z‐scores in the upper right corner, it is difficult to visually identify reasonable clusters of ID subentities. The distribution of ID and No‐ID cases visually appears similarly random in a cluster heatmap of a data set including only T cell subsets (Figure E8A); the accompanying PCA shows highly overlapping clusters (Figure E8B).

**Figure 3:**
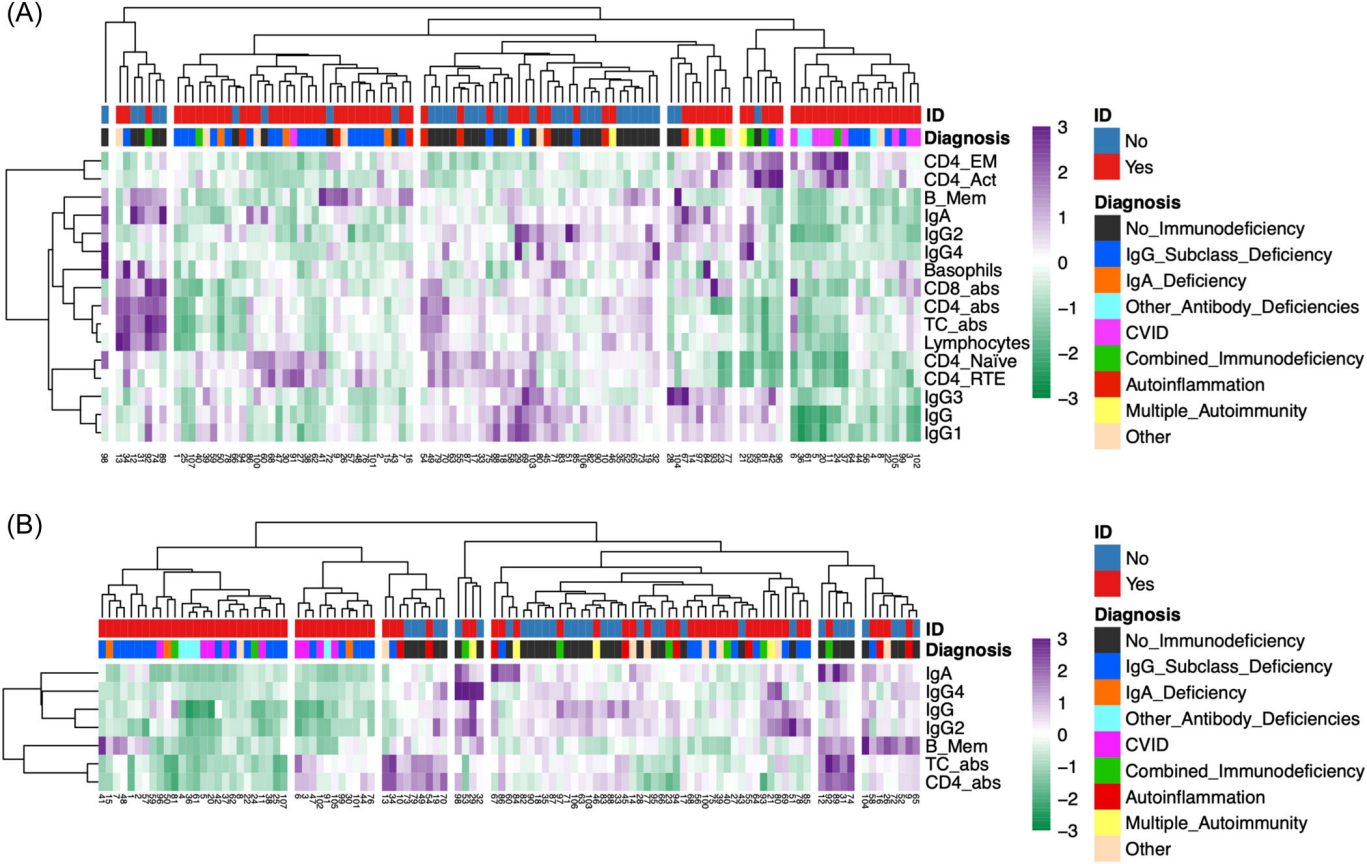
Cluster analysis of 16 moderately (A) and 7 strongly (B) differing variables between groups (107 patients): Clusters in columns dendrogram of panel A and B were selected visually according to maximum height of fusion on vertical axis. abs, absolute; CVID, common variable immunodeficiency; ID, immunodeficiency, TC, T cells. Abbreviations for B and T cell subsets see Table 1. Color scale: Z‐scores.

As k‐means clustering showed a better performance for datasets limited to moderately or strongly differing variables, hierarchical cluster heatmap for these datasets were also assessed (Figure 3A,B). Visually, clusters of ID subgroups could here be discerned. Clustering of CVID or CID cases is more apparent in Figure 3A than in Figure 3B. The cluster on the far right in Figure 3A contains 80% of CVID cases. A PCA depicting the visually chosen clusters from the column dendrogram in Figure 3A is shown in Figure E9. Clusters containing most No‐ID individuals (clusters 2 and 4) have little overlap with clusters containing predominantly individuals from the ID group.

**Figure 4:**
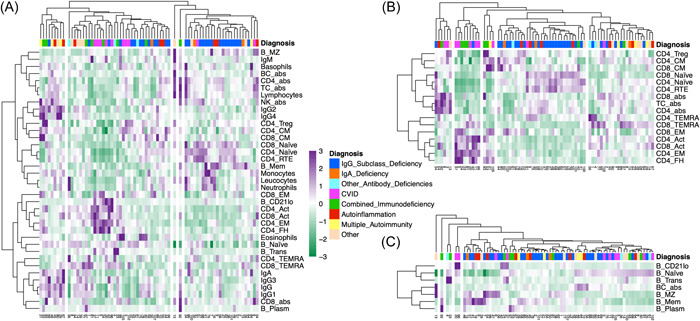
Cluster analysis of 73 patients with immunodeficiency: Cluster heatmaps including all variables (A), including only T cell variables (B) and including only B cell variables (C) are shown. The five clusters in the columns dendrograms of panel B were visually selected according to the maximum height of fusion on the vertical axis. (A PCA for these five clusters is shown as Figure E9 in the supplementary material). For panels A and C, the number of clusters was not selected visually, but was taken from panel B to allow direct comparison. abs, absolute; CVID, common variable immunodeficiency, TC, T cells. Abbreviations for T cell subsets see Table 1. Color scale: Z‐scores.

To evaluate whether a limitation to ID cases would enhance clustering of ID subentities, different sets of variables and the resulting hierarchical cluster heatmaps were again visually assessed (Figure 4A‐C). For the data set including only T cell variables, it was possible to visually identify clinically meaningful clusters (Figure 4B). For the datasets with all variables or only B cell variables, this was difficult with multiple clusters only containing 1–2 patients (Figure 4A,C). The clustering of ID subentities seems limited and at random with the data set including only B cell variables (Figure 4C). In contrast, the clustering using T cell variables only (Figure 4B) shows ID subgrouping with a large cluster of IgG subclass‐, IgA‐ and other antibody deficiencies (approx. 80% of the second cluster from the right) and two clusters consisting of CVID and CID (second and third cluster from the left). The corresponding PCA is shown in Figure E10, which sets apart two clusters containing the majority of CVID and CID patients.

Highly correlated individual variables (as identified in the correlation maps) are mostly identifiable in the multivariate clustering (rows in Figure 2), but clusters with variables that show weak individual correlations were also identified (e.g., CD4/8_CM and CD4_Treg).

## 4 DISCUSSION

In our study, we aimed to evaluate relationships between lymphocyte subsets, particularly T cells, and other immune variables and to investigate the characterization/classification of adult individuals screened for ID/PID using cluster analyses and whether this could assist clinical decision making in daily practice.

The correlation heatmaps and the cluster heatmap (Figure 2) depict some well‐established relationships of immune variables, even in the whole data set. The observed correlations with age (negative for CD4_RTE and CD4/8_Naïve, positive for CD4_Act and CD8_EM), likely reflect the thymic output of naïve T cells decreasing with age and activation of T cells associated with ageing and senescence.^25^, ^31^, ^32^ This was in keeping with a negative correlation of CD4_Treg with age in the No‐ID group.^25^, ^33^ Some of these age correlations were much weaker or even inverted within the ID group (e.g., CD4_TEMRA). An explanation of these findings might be premature senescence, chronic T cell activation or reduced thymic function enriched in ID.^31^, ^33^, ^34^, ^35^, ^36^ IgM forms a cluster with B_MZ, which are important producers of IgM in vivo, while the other immunoglobulins clustered with B_Mem.^37^ IgE levels were moderately negatively correlated to CD4_FH in the ID group, in line with elevated IgE in the absence of cognate T cell help.^38^ CD4_FH are also known producers of IL‐21, which inhibits IgE production.^39^, ^40^, ^41^ Consistently, IL‐21R deficient patients often have elevated IgE levels and loss of STAT3 signaling downstream of IL‐21R is associated with hyper IgE syndrome.^42^, ^43^, ^44^ While an expansion of CD4_FH in the specific ‘IgG4‐related disease’ entity has been described, a weak negative correlation was present in the ID group (Figure E7).^45^ CD4_FH are often expanded in peripheral blood in CVID complicated by autoimmune cytopenia, related to increased endotoxemia in these patients.^46^ A relative increase in CD4_FH in CVID patients was well appreciated in the multivariate clustering (Figure 4A,B). The clustering of CD4_FH, CD4_EM and B_CD21lo is driven by a cluster of CVID and CID with highly positive Z‐Scores in these variables (Figure 2). The correlation of CD4_FH and B_CD21lo was previously described in CVID and has been recently reviewed.^47^, ^48^ B_CD21lo were described in patients with various IEI, especially those with lymphoproliferation.^49^ Recently, it has been shown that B_CD21lo express and require the transcription factor T‐bet for their generation and are promoted by interferon‐γ.^48^, ^50^ In CVID, T and B cell dysregulations correlate with autoimmune cytopenias or polyclonal lymphoproliferation.^34^, ^51^, ^52^, ^53^, ^54^ Together with a reduction of class‐switched B_Mem and elevated CD4_FH, an elevation of B_CD21lo helped differentiate primary from secondary hypogammaglobulinemia.^55^

Multivariate analyses are suitable for assigning patients to diagnostic groups. However, prior evidence on this approach in patients with suspected PID is limited. Attardi et al. used PCA of T cell LFC in peripheral blood to compare a cohort of 100 children with PID (fewer immunoglobulin deficiencies and more severe PIDs than observed in a prototypic adult cohort) with 30 controls.^28^ Severe‐ and CID showed segregated clusters compared with healthy donors, the discriminating variables being primarily CD4_CM, CD8_EM and CD8_TEMRA. Some milder antibody defects clustered with CID, potentially identifying a more severe ID than suspected.^28^ Neirinck et al. recently performed an excellent validation of the EuroFlow PIDOT on 434 children and adults with suspected PID (318 with PID [median age 14]) and 68 healthy controls.^27^ They identified ten features (eight lymphocyte subpopulations, age, and Ig levels) being the most discriminative between the groups, seven of which we also analyzed and five of which also showed moderate evidence for a difference between groups in our analysis. PIDOT showed a sensitivity of 61% and a specificity of 60% for identifying PID in their cohort. The more than 50% of patients with predominantly antibody deficiency were difficult to identify and differentiate from disease controls with LFC only. Based on these limitations, they came up with an easy‐to‐use decision‐tree algorithm in suspected PID, which includes immunoglobulin levels and age in addition to the most discriminative lymphocyte subpopulations, resulting in an 86% sensitivity and 82% specificity (selective for lymphoid PIDs).^27^

We performed cluster analyses with two main objectives. K‐means was used to assess the proportion of correct binary differentiation into ID and Non‐ID groups. The hierarchical cluster heat maps were evaluated visually to assess whether clinically useful information could be obtained, particularly with respect to clustering of ID subclasses. While the results of k‐means could be statistically evaluated, this was not possible for a visual analysis. It is important to realize that for such a complex method to be used in day‐to‐day clinical decision making, it must be able to be performed within minutes by a physician. Visual analysis of a heat map is a feasible way to evaluate such multivariate data in clinical practice.

In our cohort of 107 individuals, k‐means cluster analysis including all 37 variables or only T cell variables did not result in a useful partition of ID and No‐ID individuals. This is in line with the results by Attardi et al., where the PCA of T cell subpopulations was unable to differentiate CVID from healthy donors.^28^ Similar to the study by Neirinck et al., the limitation to differing variables between groups improved the binary segregation with correct classification of approximately 75% of patients if only strongly differing variables were assessed, compared to 54% with the whole data set.^27^ Applying the test characteristics of the PIDOT‐algorithm to our population would have resulted in 85% correct classifications (60.7% if only the PIDOT tube alone was used). Because we did not exclusively include lymphoid PIDs, this is likely an overestimate.^27^ Considering that most patients in our cohort displayed rather mild immunoglobulin deficiencies, the observed proportion of correct classifications is considerable. We would expect an even higher proportion if the population contained more severe PIDs such as CVID or CID. Because many patients with IgG subclass deficiency display normal lymphocyte distributions and are difficult to differentiate from immunocompetent individuals by LFC alone, a low proportion of correct binary classification with a data set containing only T cell variables was expected. Similarly, the diagnostic accuracy was very limited when only the PIDOT was used in the study by Neirinck et al.^27^

By visual analysis of the cluster heatmap of the whole data, not much information could be gained. Thus, the clinical usefulness of a heatmap with the full data set seems limited. A more nuanced picture emerged for the cluster heatmaps with the optimized datasets including moderately or strongly differing variables. Especially the former allowed the rapid identification of most CVID or CID patients in the same clusters (Figure 3A). While the binary classification of ID *versus* No‐ID with k‐means was better for the data set including only seven strongly differing variables, the clustering of the more severe ID subentities is most apparent in the data set including 16 moderately differing variables, including six T cell subsets. This could possibly indicate that for a reliable identification of different ID subentities, the inclusion of moderately differing variables is superior to limiting the data set only to the most strongly differing variables.

For the cluster analysis within the ID group (Figure 4), we could observe meaningful clustering for the data set including 16 T cell variables, but not for the data set with all variables or B cell restricted variables. Especially the more significant IDs, CVID and CID, clustered separately in the former (Figure 4B, Figure E10B). The segregation of most CID with CVID reinforces the concept of late‐onset combined immunodeficiency, which amongst other features is associated with increased morbidity and reduced survival.^56^ Two patients in our cohort with the clinical diagnosis of IgG subclass deficiency displayed a remarkably similar T cell distribution to the patients with CVID or CID (first two blue bars from the left in Figure 4B), which will enforce clinical revisitation.

While in our cohort, the inclusion of T cell subpopulations did not result in a significant improvement in the correct allocation to the No‐ID or ID groups, this may be different in cohorts with more severe IDs. For example, pediatric cohorts or PID cohorts evaluated for allogenic stem cell transplantation may include more CID. Six T cell subsets were included in the moderately differing variable data set which resulted in the most helpful cluster heatmap for the whole population (Figure 3A). Also, the heatmap for the ID group including only T cell variables contained the clinically most meaningful clustering (Figure 4B). This may suggest that for a clustering approach in suspected PID, LFC panels with T cell subclasses are preferable. This is supported by the PIDOT‐algorithm which also includes two T cell subsets (CD4_Naïve, total CD4+ T cells).

Our study has several limitations. The No‐ID group consisted of individuals referred to the immunodeficiency clinic for a clinical reason, usually due to apparent susceptibility to infections. While healthy blood donors as a comparison group is a valid alternative approach, our control group better represents the real‐life situation in the clinic. Our approach has been used for similar studies.^27^ In addition, an ‘initial’ clinical diagnosis is a cross‐sectional process and may need to be revised over time as new clinical events, such as severe or opportunistic infections, occur. Also, patients susceptible to infections due to innate immune defects may have normal LFC results and immunoglobulins and may be misclassified. Therefore, some of the “misclassifications” by cluster analysis may be due to an incorrect clinical diagnosis. Although clinical diagnosis, according to the ESID definitions, is inevitably imperfect as a ‘reference standard’, it seems to be the most appropriate choice, which is supported by its use in other studies.^27^ The number of patients for PID sub‐entities, apart from IgG subclass deficiency and CVID, was too small for a meaningful subclass analysis. Also, the clinical umbrella of IgG subclass deficiency is heterogenous in terms of molecular/genetic pathogenesis and clinical prognosis. Attempts to study IgG subclass deficiency in more detail in multicenter cohorts have been initiated.^57^ Most patients in our ID group had antibody deficiency, whereas combined or severe combined PID/IEI were underrepresented compared to pediatric cohorts. Also, pure phagocyte or complement disorders are IEI which are typically missed if LFC is performed exclusively during the diagnostic process. A minority in our ID group had a diagnosis of secondary ID. It is increasingly demonstrated that patients with secondary ID may actually have an underlying IEI. E.g., lymphoma may occur as a complication of CVID rather than being its cause.^3^ As these patients were regularly evaluated in the ID outpatient clinic for the presence of an PID/IEI, we did not exclude them from the main analysis. Importantly, the k‐means cluster analysis showed no relevant difference with and without these patients (Table 2).

The PIDOT has been developed and validated as a diagnostic test for suspected PID and can be applied in different centers and is a preferred method in the evaluation for suspected PID.^27^ However, the PIDOT has not been implemented in many centers, including ours. Thus, a cluster approach as described here may support clinical decision making. Individual centers could compile their own datasets of ID versus No‐ID patients, possibly including additional variables such as complement factors as well, and use open‐source software (e.g., Clustvis) to perform clusters analysis within minutes without specialized statistical knowledge.^58^ By indexing the values of the variables, for example, to the center‐specific lower limit of the reference range, results from different centers could be pooled and reference datasets, ideally from genetically assessed pediatric and/or adult populations, could be made publicly available. Visual inspection of heat maps and the localization of the individual under investigation may facilitate the interpretation of multiple immune variables simultaneously and assist in correct clinical diagnosis (ID vs. No‐ID) but also advice, whether additional diagnostic and/or genetic testing may be indicated. In case of a clinical diagnosis of an ID without an exact genetic diagnosis (which is very common in adult patients) the position of this individual in a heat map containing patients with known ID subentities could facilitate diagnosis of the correct PID subentity. Prospective studies are currently planned to investigate whether such an approach is beneficial.

In summary, multivariate cluster analysis of a reduced set of immune variables including T‐cell subsets, correctly predicted an individual's immune status (ID vs. No‐ID) in approximately 75% and allowed for visual identification of more severe subentities within the ID group, such as CVID or CID. In keeping with results from other groups, our study supports the use of detailed LFC, including T cell subpopulations, in the work‐up for suspected PID.^17^, ^27^, ^28^ This approach is also reflected by the current ESID working definition for the clinical diagnosis of CVID, where total CD4+ and CD4_Naïve need to be assessed.^59^, ^60^ With the use of online open‐source software and a reference data set, cluster analysis may be performed within minutes in the outpatient clinic.

## AUTHOR CONTRIBUTIONS

**Luca Seitz**: Conceptualization; Data curation; Formal analysis; Funding acquisition; Investigation; Methodology; Project administration; Software; Validation; Visualization; Writing—original draft; Writing—review & editing. **Daniel Gaitan**: Conceptualization; Data curation; Investigation; Writing—review & editing. **Caroline M Berkemeier**: Validation; Visualization; Writing—review & editing. **Christoph T Berger**: Validation; Writing—review & editing. **Mike Recher**: Conceptualization; Data curation; Funding acquisition; Methodology; Project administration; Resources; Supervision; Writing—review & editing.

## CONFLICT OF INTEREST STATEMENT

The authors declare no conflict of interest.

## ETHICS STATEMENT

The study was approved by the local institutional review board (Ethics Committee Nordwest‐ und Zentralschweiz, Switzerland), approval number 2017‐02029.

## Supporting information

Supporting information.

## Data Availability

The data that support the findings of this study are available from the corresponding author upon reasonable request.
