# Supplementary material for: Cluster analysis of flowcytometric immunophenotyping with extended T cell subsets in suspected immunodeficiency
Source: Immun Inflamm Dis. 2023 Dec 6;11(12):e1106. doi: 10.1002/iid3.1106 (PMC10698832; doi:10.1002/iid3.1106)

**Supplementary Material**

**Table E1. Data overview for individual variables and both patient groups**

BC, B cells; CD, cluster of differentiation; NK, natural killer cells; TC, T cells

|  | | | **No Immunodeficiency** | | | | | | **Immunodeficiency** | | | | | |
| --- | --- | --- | --- | --- | --- | --- | --- | --- | --- | --- | --- | --- | --- | --- |
|  | **reference**  **range** | **unit** | **n** | **mean** | **sd** | **median** | **Q_1_** | **Q_3_** | **n** | **mean** | **sd** | **median** | **Q_1_** | **Q_3_** |
| **Age** | - | years | 34 | 43.5 | 14.6 | 42.0 | 33.5 | 53.0 | 73 | 45.6 | 16.0 | 47.0 | 35.0 | 57.0 |
| **TC_%** | 55 - 86 | % lymphocytes | 34 | 77.6 | 5.3 | 77.5 | 75.0 | 82.0 | 73 | 73.5 | 9.8 | 75.0 | 68.0 | 80.0 |
| **TC_absolute** | 742 - 2750 | / µl | 34 | 1855.9 | 558.6 | 1734 | 1500 | 2166 | 73 | 1482.4 | 625.1 | 1473 | 1087 | 1747 |
| **CD4_%** | 33 - 58 | % lymphocytes | 34 | 50.2 | 7.2 | 49.5 | 47.0 | 54.0 | 73 | 45.6 | 11.8 | 47.0 | 38.0 | 53.0 |
| **CD4_absolute** | 404 - 1612 | / µl | 34 | 1211.5 | 385.4 | 1175.5 | 918.5 | 1435.5 | 73 | 916.4 | 424.3 | 867 | 619 | 1221 |
| **CD4_Naïve_%** | 15.7 - 54.7 | % CD4+ TC | 34 | 40.1 | 14.2 | 39.3 | 28.7 | 48.8 | 73 | 31.4 | 16.2 | 31.6 | 22.5 | 43.2 |
| **CD4_Central_Memory_%** | 8.0 - 28.9 | % CD4+ TC | 34 | 25.5 | 6.4 | 25.8 | 22.3 | 28.9 | 73 | 23.8 | 7.2 | 24.6 | 19.0 | 28.2 |
| **CD4_Effector_Memory_%** | 16.8 - 57.4 | % CD4+ TC | 34 | 29.9 | 11.7 | 29.3 | 19.5 | 37.3 | 73 | 39.2 | 17.8 | 34.6 | 25.5 | 47.6 |
| **CD4_Terminally_Differentiated_%** | 3.6 - 23.2 | % CD4+ TC | 34 | 5.3 | 3.5 | 4.4 | 3.6 | 5.9 | 73 | 5.7 | 3.8 | 4.8 | 3.5 | 6.7 |
| **CD4_Recent_Thymic_Emigrants_%** | 14.1 - 37.2 | % CD4+ TC | 34 | 28.3 | 10.0 | 28.2 | 21.6 | 33.8 | 73 | 23.5 | 13.4 | 21.2 | 14.9 | 31.6 |
| **CD4_Follicular_Helper_%** | 6.9 - 19.1 | % CD4+ TC | 34 | 12.3 | 4.0 | 11.4 | 9.7 | 15.0 | 73 | 15.8 | 8.8 | 12.9 | 11.1 | 18.0 |
| **CD4_Regulatory_T Cell_%** | 6.1 - 11.0 | % CD4+ TC | 34 | 7.4 | 2.0 | 7.3 | 6.3 | 8.4 | 73 | 7.2 | 2.6 | 7.2 | 6.2 | 7.9 |
| **CD4_Activated_%** | 4.1 - 15.6 | % CD4+ TC | 34 | 5.6 | 4.5 | 4.9 | 3.5 | 6.2 | 73 | 8.0 | 5.4 | 5.9 | 4.1 | 11.4 |
| **CD8_%** | 13 - 39 | % lymphocytes | 34 | 26.6 | 7.1 | 27.0 | 21.3 | 31.0 | 73 | 27.0 | 10.9 | 24.0 | 21.0 | 33.0 |
| **CD8_absolute** | 220 - 1129 | / µl | 34 | 636.0 | 250.3 | 646 | 472.2 | 745.5 | 73 | 540.2 | 298.0 | 468 | 344 | 698 |
| **CD8_Naïve_%** | 7.0 - 62.5 | % CD8+ TC | 34 | 35.3 | 20.5 | 35.7 | 16.9 | 41.7 | 73 | 27.4 | 19.2 | 24.7 | 11.2 | 44.6 |
| **CD8_Central_Memory_%** | 0.6 - 4.4 | % CD8+ TC | 34 | 5.4 | 4.8 | 4.2 | 2.9 | 5.4 | 73 | 4.7 | 3.7 | 3.9 | 2.0 | 6.0 |
| **CD8_Effector_Memory_%** | 4.3 - 64.5 | % CD8+ TC | 34 | 32.5 | 14.9 | 31.5 | 21.4 | 40.9 | 73 | 35.0 | 14.5 | 33.8 | 24.7 | 43.8 |
| **CD8_Terminally_Differentiated_%** | 8.1 - 60.5 | % CD8+ TC | 34 | 26.8 | 12.2 | 26.9 | 18.8 | 36.9 | 73 | 33.0 | 19.9 | 27.9 | 16.7 | 47.1 |
| **CD8_Activated_%** | 8.7 - 45.2 | % CD8+ TC | 34 | 16.9 | 13.8 | 14.4 | 6.8 | 22.1 | 73 | 18.5 | 13.7 | 13.8 | 9.7 | 26.4 |
| **NK_%** | 5 - 26 | % lymphocytes | 34 | 9.7 | 4.6 | 9.0 | 6.0 | 12.0 | 73 | 10.9 | 6.3 | 10.0 | 8.0 | 13.0 |
| **NK_absolute** | 84 - 724 | / µl | 34 | 222.6 | 103.6 | 217.0 | 134.5 | 286.2 | 73 | 205.7 | 113.9 | 195 | 124 | 259 |
| **BC_%** | 5 - 22 | % lymphocytes | 34 | 11.8 | 4.4 | 12.0 | 9.0 | 14.0 | 73 | 14.9 | 9.1 | 13.0 | 9.0 | 18.0 |
| **BC_absolute** | 80 - 616 | / µl | 34 | 281.1 | 137.5 | 268.5 | 189.2 | 352.0 | 73 | 323.5 | 388.4 | 239 | 147 | 372 |
| **BC_Naïve_%** | 25.1 - 92.4 | % B cells | 33 | 63.0 | 13.7 | 66.1 | 53.7 | 71.6 | 70 | 67.9 | 20.7 | 70.9 | 57.1 | 84.2 |
| **BC_Marginal_Zone_Like_%** | 3.1 - 59.7 | % B cells | 33 | 16.9 | 9.0 | 14.9 | 9.1 | 22.1 | 70 | 17.7 | 15.7 | 13.5 | 6.8 | 23.5 |
| **BC_Memory_%** | 2.4 - 32.6 | % B cells | 33 | 13.9 | 6.7 | 12.3 | 8.4 | 18.7 | 70 | 9.0 | 7.3 | 7.5 | 3.5 | 12.8 |
| **BC_CD21low_%** | 0.5 - 4.7 | % B cells | 33 | 1.5 | 1.1 | 1.1 | 0.8 | 1.8 | 70 | 2.4 | 4.4 | 1.1 | 0.7 | 2.5 |
| **BC_Transitional_%** | 0.3 - 2.9 | % B cells | 33 | 2.0 | 1.1 | 2.0 | 1.1 | 2.7 | 70 | 2.9 | 4.8 | 2.0 | 1.2 | 2.9 |
| **BC_Plasmablast_%** | 0.1 - 3.0 | % B cells | 33 | 0.6 | 0.7 | 0.3 | 0.2 | 0.6 | 70 | 0.8 | 2.2 | 0.2 | 0.1 | 0.6 |
| **Leucocytes** | 3.5 - 10.0 | * 10^9 / L | 34 | 6.69 | 1.97 | 6.57 | 5.25 | 7.92 | 71 | 6.94 | 2.61 | 6.56 | 4.94 | 8.45 |
| **Neutrophils** | 1.3 - 6.7 | * 10^9 / L | 33 | 3.88 | 1.54 | 3.68 | 2.95 | 4.57 | 69 | 4.33 | 2.17 | 3.94 | 3.03 | 5.10 |
| **Lymphocytes** | 0.9 - 3.3 | * 10^9 / L | 33 | 2.10 | 0.56 | 1.99 | 1.73 | 2.45 | 69 | 1.82 | 0.83 | 1.73 | 1.25 | 2.13 |
| **Monocytes** | 0.12 - 0.62 | * 10^9 / L | 33 | 0.37 | 0.15 | 0.32 | 0.27 | 0.44 | 69 | 0.38 | 0.13 | 0.36 | 0.29 | 0.45 |
| **Eosinophils** | 0 - 0.30 | * 10^9 / L | 33 | 0.18 | 0.15 | 0.15 | 0.11 | 0.20 | 69 | 0.15 | 0.11 | 0.11 | 0.08 | 0.17 |
| **Basophils** | 0 - 0.09 | * 10^9 / L | 33 | 0.05 | 0.02 | 0.05 | 0.03 | 0.05 | 69 | 0.04 | 0.02 | 0.03 | 0.03 | 0.05 |
| **IgG** | 7.0 - 16.0 | g / L | 33 | 11.65 | 1.85 | 11.50 | 10.00 | 12.90 | 71 | 9.06 | 3.92 | 8.60 | 6.66 | 12.20 |
| **IgG1** | 4.9 - 11.4 | g / L | 24 | 7.28 | 1.43 | 7.22 | 6.32 | 7.87 | 57 | 5.83 | 2.58 | 5.46 | 3.81 | 7.70 |
| **IgG2** | 1.5 - 6.4 | g / L | 24 | 3.64 | 1.27 | 3.51 | 2.91 | 3.95 | 56 | 2.32 | 1.64 | 2.06 | 1.12 | 3.31 |
| **IgG3** | 0.2 - 1.1 | g / L | 24 | 0.48 | 0.29 | 0.40 | 0.28 | 0.53 | 56 | 0.36 | 0.25 | 0.29 | 0.18 | 0.45 |
| **IgG4** | 0.08 - 1.4 | g / L | 24 | 0.86 | 0.71 | 0.82 | 0.40 | 1.00 | 55 | 0.34 | 0.50 | 0.22 | 0.05 | 0.37 |
| **IgA** | 0.7 - 4.0 | g / L | 33 | 2.24 | 1.08 | 1.90 | 1.63 | 2.40 | 67 | 1.37 | 1.11 | 1.25 | 0.42 | 2.03 |
| **IgM** | 0.4 - 2.3 | g / L | 33 | 1.22 | 0.93 | 1.10 | 0.75 | 1.29 | 67 | 1.36 | 1.66 | 0.87 | 0.55 | 1.50 |
| **IgE** | < 100 | IU / ml | 25 | 1119.3 | 5184.85 | 29.00 | 13.00 | 100.00 | 42 | 29.99 | 69.58 | 14.00 | 2.50 | 31.50 |

**Table E2. Box-Plots (including bee-swarm) of 43 laboratory variables and age**

Scales on Y-axis are linear (exceptions with logarithmic scale: “B cells CD21 low”, “B cells Transitional”, “B cells Plasmablasts”, “Ig4”, “IgM” and “IgE”). Grey horizontal dotted lines are reference ranges from the local laboratory. Boxes showing median and interquartile range. P-values according to Wilcoxon ranks sum test.

|  |  |  |
| --- | --- | --- |
|  |  |  |
|  |  |  |
|  |  |  |
|  |  |  |
|  |  |  |
|  |  |  |
|  |  |  |
|  |  |  |
|  |  |  |
|  |  |  |
|  |  |  |
|  |  |  |
|  |  |  |
|  |  |  |
|  |  |  |

**Table E3. Comparison of the proportion of correct binary grouping of patients with and without immunodeficiency by k-means and k-medoids clustering methods.**

| **Patients included** | **Variables included** | **Correctly classified**  **k-means** | **Correctly classified**  **k-medoid** |
| --- | --- | --- | --- |
| All 107 | All 37 variables | 54.2% | 57.0% |
| All 107 | 20 (with weak evidence for difference between groups (p<0.1) | 57.0% | 56.0% |
| All 107 | 16 (with moderate evidence for difference between groups (p<0.05)) | 69.1% | 56.0% |
| All 107 | 10 (with moderate evidence for difference between groups (p<0.05), no T cell subsets) | 68.2% | 68.2% |
| All 107 | 7 (with strong evidence for difference between groups (p<0.01)) | 74.8% | 71.0% |
| All 107 | 5 (with strong evidence for difference between groups (p<0.01), no T cell subsets) | 73.8% | 73.8% |
| All 107 | 16 (only T cell variables) | 53.3% | 58.9% |
| 100 (no secondary) | All 37 variables | 53.0% | 58.0% |
| 100 (no secondary) | 7 (with strong evidence for difference between groups (p<0.01)) | 76.0% | 73.0% |

**Supplementary Text E1: Correlations of T cell subsets with each other and with other immune system laboratory parameters** (Figures E1-6 below)

**Figure E2** shows the correlation of T cell subsets with each other. As expected, a very strong positive correlation between CD4_RTE and CD4_Naïve for both groups and a strong to very strong negative correlation between CD4_EM and CD4_Naïve/CD4_RTE were found. Also expectedly, CD4_Naïve show a negative correlation with CD4_Act while CD4_Naïve show a moderate to strong positive correlation with CD8_Naïve. CD4_Act demonstrate a very strong positive correlation with CD8_Act. Peculiarly, CD4_Treg had only weak correlations with other T cell subpopulations. Apart from minor differences, the T cell subset correlations were very similar in the two diagnostic groups (No-ID and ID). There were however some exceptions: Very weak positive correlations of CD4_Naïve with CD8_CM were found in the ID while a weak negative correlation was computed in the No-ID group. Also, CD8_EM demonstrated marked differences of correlation coefficients when plotted against multiple other T cell subsets, mostly with smaller correlation coefficients in the ID group.

**Figure E3** shows the correlation of T cell subsets with the three main lymphocyte populations in peripheral blood (T cells *vs.* B cells or NK cells). Apart from the expected strong to very strong positive correlation of absolute numbers of CD4+ and CD8+ T cells with absolute numbers of T cells and lymphocytes, mostly weak correlations were found. An exception was the absolute number of B cells, which showed a weak to moderate positive correlation to absolute numbers of CD4/8+ T cells within the ID group. There was also a notable difference in correlation of percentage/absolute numbers of NK cells with CD4/8_Act and CD4/8_TEMRA between groups, with a weak positive correlation in the No-ID and a very weak to weak negative correlation in the ID group.

**Figure E4** shows the correlation of T cell subsets with B cell subsets. For B_Mem, there were divergent correlations for multiple parameters between diagnostic groups. B_Mem weakly to moderately negatively correlated with CD4_Naïve, CD4_RTE and CD8_Naïve in the No-ID group, while weak positive correlations were found in the ID group. B_Mem correlated positively with CD4_EM, CD4_Act and CD8_Act in the non-ID group, while negative correlations were computed for the ID group. B_CD21lo correlated weakly negatively with CD4_Treg in the ID group.

**Figure E5** shows the correlation of T cell subsets with myeloid cells. A weak to moderate positive correlation of basophils, eosinophils, monocytes and neutrophils with absolute CD4+ T cells was found, and the correlation was larger in the No-ID *vs.* the ID group. CD4_EM, CD4_TEMRA, CD4_FH, CD4_Act and CD8 (%) showed a weak negative correlation with neutrophils for both diagnostic groups.

**Figure E6 and E7** show the correlation of T cell subsets with immunoglobulin levels and IgG subclasses. There was a weak to moderate positive correlation of IgG with CD4_EM, CD4_Act and CD8_Act within the ID group, while very weak correlations were documented for the No-ID group. IgE levels were moderately negatively correlated to CD4_FH in the ID group. IgM was moderately negatively correlated to CD4 T cells (%) and moderately positively correlated to CD4_CM. Correlations of IgG subclasses *vs.* T cells subsets were very weak to weak.

**Figure E1. Sequential gating strategy for characterization of T cell subpopulations**

Single events were identified using FSC-H/FSC-A profile. Further analysis of lymphocytes was done using a combination of CD45 expression and SSC/FSC properties. Within the CD45+ cells, T cells were defined as CD3+ cells **(A)** and subdivided into CD4+ and CD8+ T cells **(B)** based on the expression of the respective markers. Presence of CCR7, CD45RA or CD45RO was used to further separate CD8+ and CD4+ T cells into the following T cell subpopulations; CD8_Naïve (CD45RA+, CD45RO-, CCR7+), CD8_CM (CD45RA-, CD45RO+, CCR7+), CD8_EM (CD45RA-, CD45RO+, CCR7-), CD8_TEMRA (CD45RA+, CD45RO-, CCR7-) **(C)** and CD4_Naïve (CD45RA+, CD45RO-, CCR7+), CD4_CM (CD45RA-, CD45RO+, CCR7+), CD4_EM (CD45RA-, CD45RO+, CCR7-), CD4_TEMRA (CD45RA+, CD45RO-, CCR7-) **(D)**. CD4_RTE were identified by surface expression of CD31 (CD45RA+ CD45RO-, CD31+) **(E)**, expression profiles of CD25 and CD127 on CD4+ T cells were used for identification of CD4_Treg (CD25+ CD127 low) **(F)**, and presence of CXCR5 was used to define CD4_FH (CD45RA-, CD45RO+, CXCR5+) **(G)**. Whenever CD45RA or CD45RO were not directly used for identification of the individual T cell subpopulation, presence or absence of the respective marker was analyzed by back-gating of the respective T cells.

**
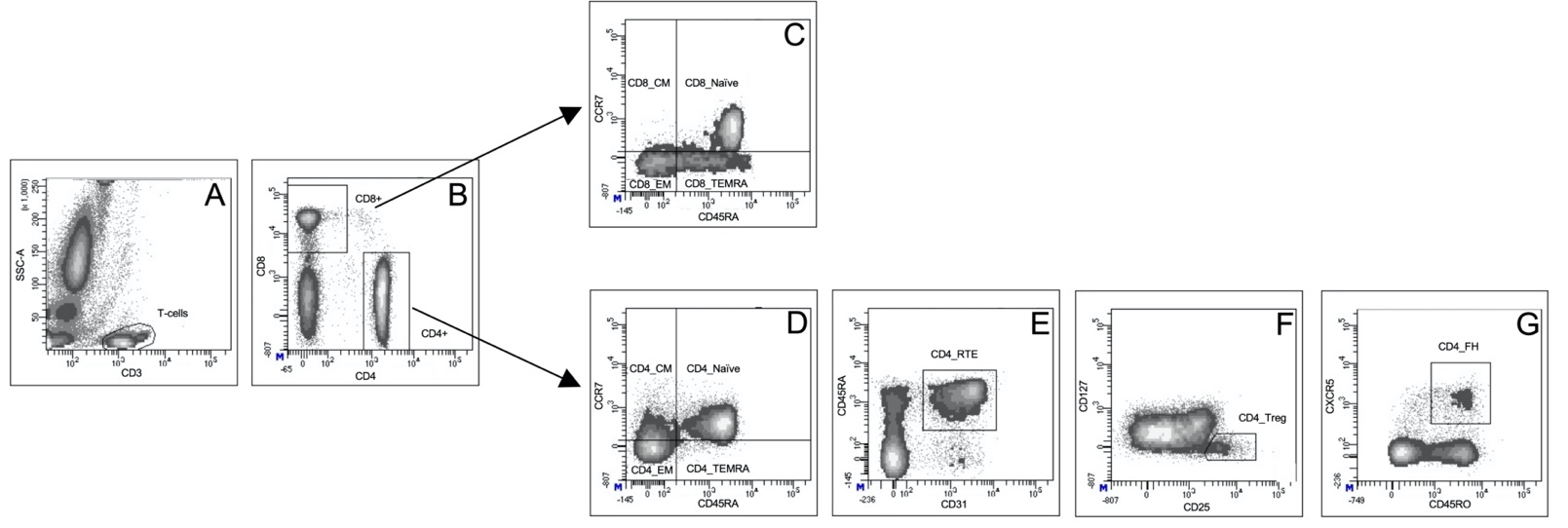
**

**Figure E2. Correlation heatmap of T cell subsets**

abs, absolute; ID, immunodeficiency. Abbreviations for T cell subsets see Table 1 (main text). Color scale: Spearman’s correlation coefficient r (-1 to 1). No missing values.

**Figure E3. Correlation heatmap of T cell subsets vs. main lymphocytes**

abs, absolute; BC, B cells; ID, immunodeficiency; NK, Natural killer cells; Lympho, lymphocytes; TC, T cells. Abbreviations for T cell subsets see Table 1 (main text). Color scale: Spearman’s correlation coefficient r (-1 to 1). Missing values: 5/2568.

**Figure E4. Correlation heatmap of T cell vs. B cell** **subsets**

abs, absolute, ID, immunodeficiency. Abbreviations for T and B cell subsets see Table 1 (main text). Color scale: Spearman’s correlation coefficient r (-1 to 1). Missing values: 24/2461.

**Figure E5. Correlation heatmap of T cell subsets vs. myeloid cells**

abs, absolute; Baso, basophils; Eosino, eosinophils; ID, immunodeficiency; Mono, monocytes; Neutro, neutrophils. Abbreviations for T cell subsets see Table 1 (main text). Color scale: Spearman’s correlation coefficient r (-1 to 1).

Missing values 20/2247.

**Figure E6. Correlation heatmap of T cell subsets vs. serum immunoglobulin levels**

abs, absolute; ID, immunodeficiency; Ig, immunoglobulin. Abbreviations for T cell subsets see Table 1 (main text). Color scale: Spearman’s correlation coefficient r (-1 to 1). Missing values 57/2247.

**Figure E7. Correlation heatmap of T cell subsets vs. IgG subclasses**

abs, absolute; ID, immunodeficiency; Ig, immunoglobulin. Abbreviations for T cell subsets see Table 1 (main text). Color scale: Spearman’s correlation coefficient r (-1 to 1). Missing values 108/2247.

**Figure E8. Cluster analysis of 16 T cell variables for 107 patients**

**A)** **Cluster heatmap:** Dendrogram of columns showing 4 clusters. abs, absolute; ID, immunodeficiency. Other abbreviations see Table 1 (main text). Color scale (green – purple): Z-scores.

**
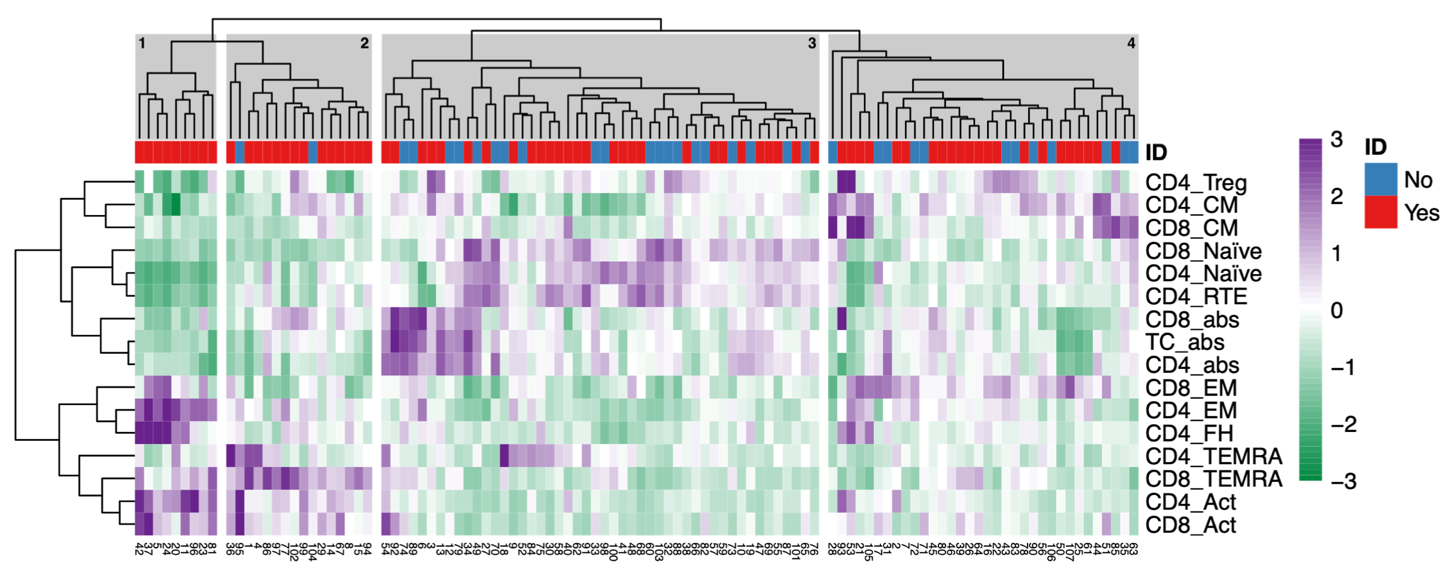
**

**B)** **PCA**: Showing the 4 clusters of the column dendrogram of Figure E7A (numbering of clusters according to numbering of Figure E7A)

**
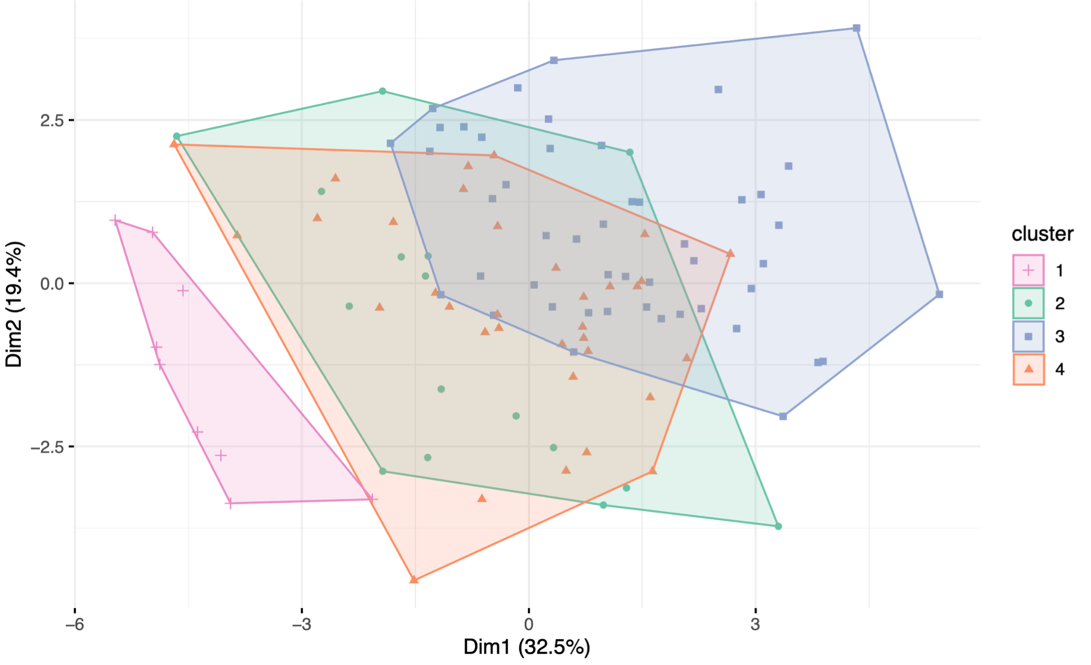
**

**Figure E9. Cluster analysis of 16 moderately differing variables between groups (107 patients).**

**A)** Clusters in columns dendrogram of panel A were selected visually according to maximum height of fusion on vertical axis. **B)** Principal components analysis of clusters selected in panel A. (Cluster 1 contains only one patient, visualization not possible; numbering of clusters according to numbering of panel A). Abbreviations: abs, absolute; CVID, common variable immunodeficiency; ID, immunodeficiency; TC, T cells. Abbreviations for B and T cell subsets see Table 1. Color scale: Z-scores.


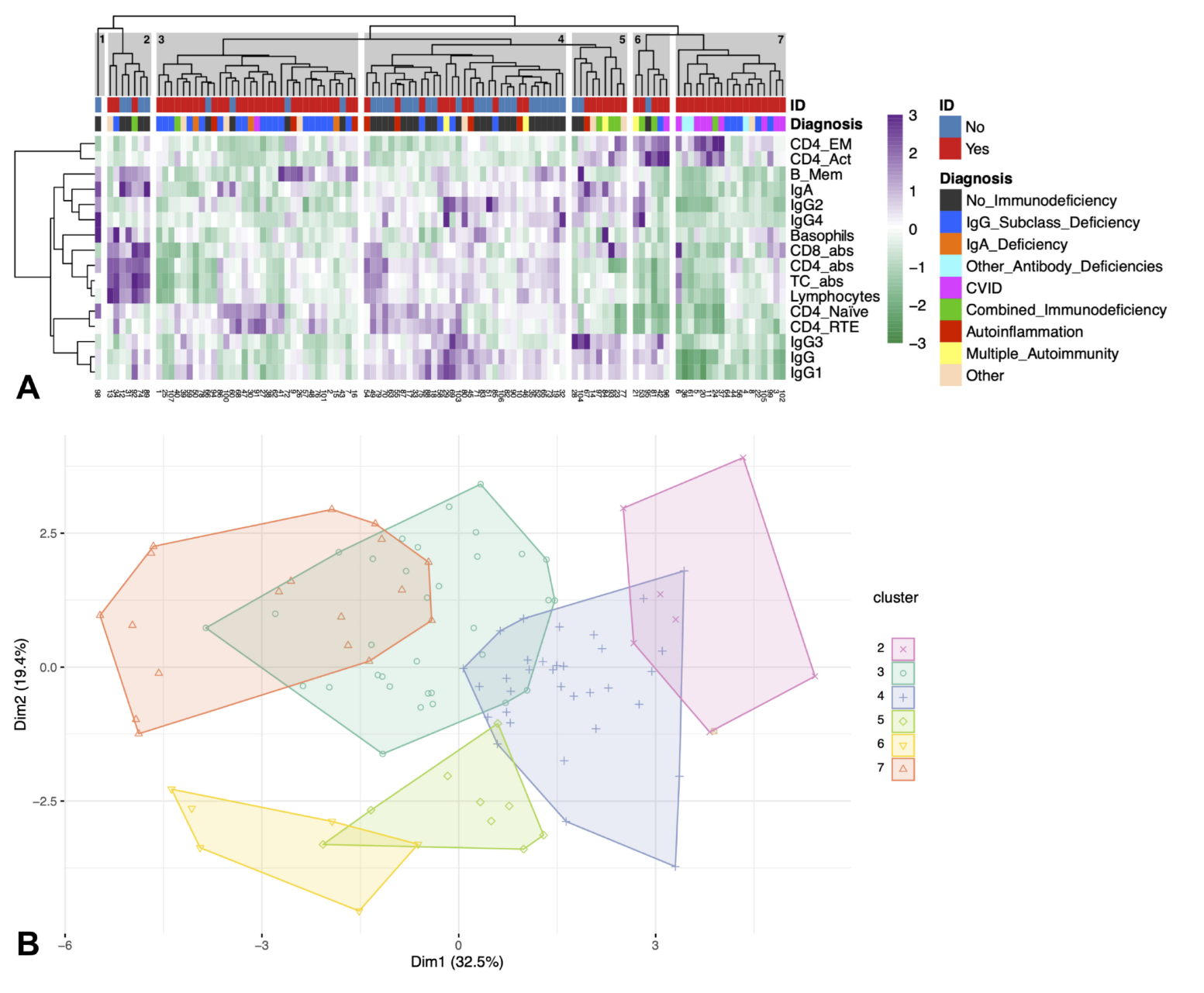


**Figure E10. Cluster analysis of 73 patients with immunodeficiency, including only T cell variables.**

**A)** Cluster heatmap: The five clusters in the columns dendrograms were visually selected according to the maximum height of fusion on the vertical axis. **B)** Principal component analysis (PCA) for the five clusters selected visually in panel A (numbering of clusters according to numbering of panel A). Abbreviations see Table 1 (main text). Color scale (green – purple): Z-scores.


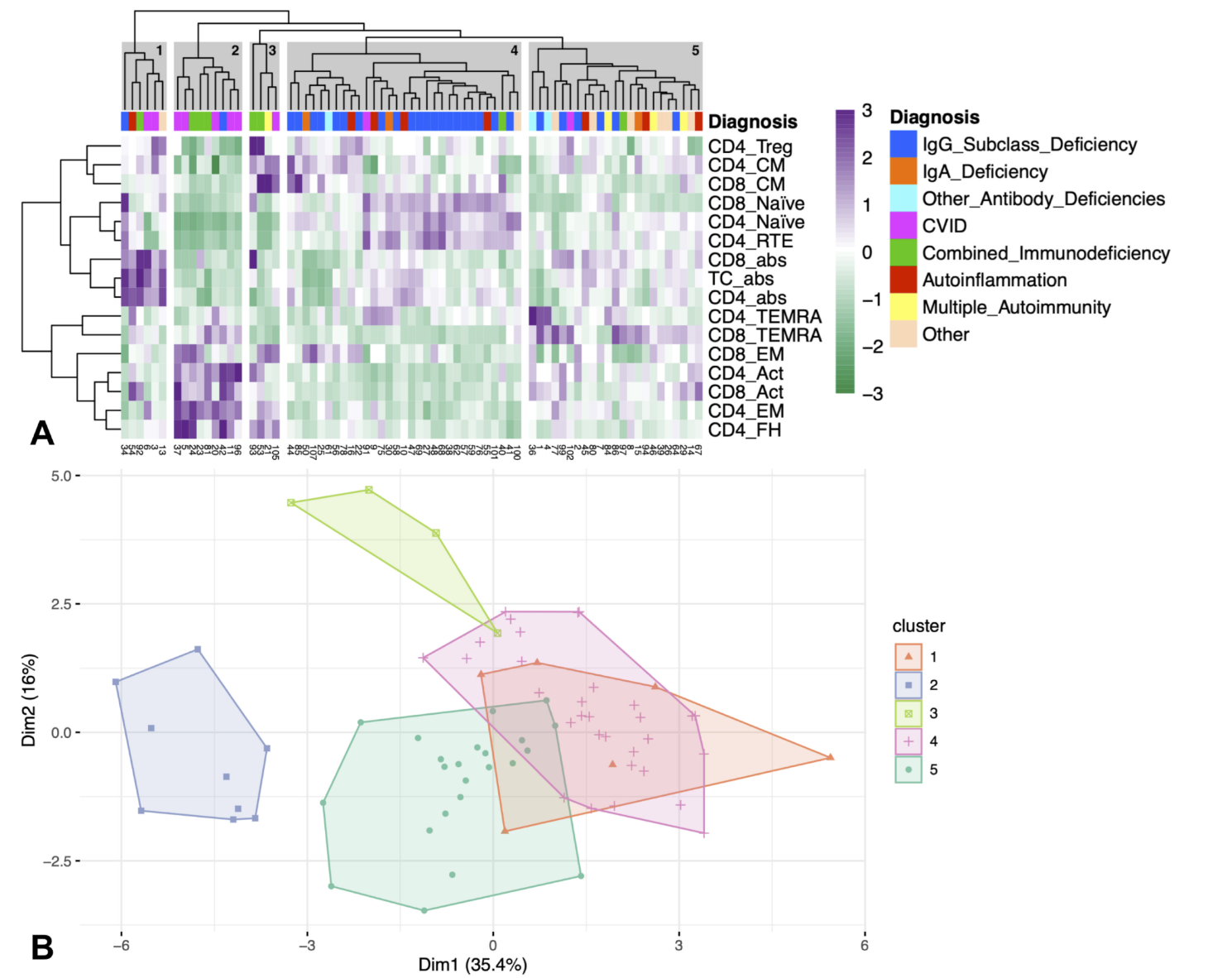

Supplement: Supplementary file 1 — Supporting information. [file IID3-11-e1106-s001.docx]
